# Effect of drying methods on yield, physicochemical properties, and total polyphenol content of chamomile extract powder

**DOI:** 10.3389/fphar.2022.1003209

**Published:** 2022-11-02

**Authors:** Sin Yee Lee, Vincent Ferdinand, Lee Fong Siow

**Affiliations:** School of Science, Monash University Malaysia, Subang Jaya, Malaysia

**Keywords:** chamomile extract powder, convection oven-drying, spray-drying, freeze-drying, total polyphenol content

## Abstract

Chamomile (*Matricaria chamomilla* L.) is a traditional medicinal plant used to treat hay fever, inflammation, muscle spasms, menstrual disorders, insomnia ulcers, wounds, gastrointestinal disorders, rheumatic pain, and hemorrhoids. Dried chamomile flowers have a longer shelf life and the dried extract in form of powder offers much flexibility for new therapeutic formulations as it could be used as a replacement for liquid extract and serve as a shelf-stable ingredient in new applications. This study aims to determine the effect of drying methods, i.e., convection oven-drying at 45 °C, freeze-drying at −50°C, and spray-drying at 140°C at 10.5 and 12 ml/min, respectively) on powder yield, physicochemical properties (moisture content, water activity, and color attributes), and total polyphenol content of chamomile extract powder. Our findings showed that spray-drying conducted at 140°C, 12 ml/min resulted in the lowest yield of powder (16.67%) compared to convection oven-drying (90.17%) and freeze-drying (83.24%). Decreasing the feed flow rate to 10.5 ml/min during spraying caused an increase in powder yield to 26.99%. The moisture content of spray-dried chamomile extract powder obtained at 140°C, 10.5 ml/min was higher (11.00%) compared to that of convection oven-dried (8.50%) and freeze-dried (7.50%). Both convection oven-dried and freeze-dried chamomile extract powder displayed no significant difference (*p* > 0.05) in moisture content. The higher feed flow rate (12 ml/min) in spray-drying also led to an increase in the moisture content of chamomile extract powder to 12.00%. The higher residual moisture found in the spray-dried samples resulted in partial agglomeration of particles. In terms of water activity, freeze-dried chamomile extract powder was found to have the highest water activity (0.63) compared to that of convection oven-dried (0.52), spray-dried at 140°C, 10.5 ml/min (0.57), and spray-dried at 140°C, 12 ml/min (0.58). Spray-dried and freeze-dried chamomile extract powder with high moisture content and water activity could be highly susceptible to microbial growth. In terms of color attributes, higher drying temperature in spray-drying led to darker, redder, and more yellowish chamomile extract powder that could be caused by heat-induced Maillard reaction and caramelization. Since lower drying temperature was used in both convection oven-drying and freeze-drying, both convection oven-dried (56.94 mg GAE/g powder) and freeze-dried chamomile extract powder (55.98 mg GAE/g powder) were found to have higher total polyphenol content compared to those of spray-dried (42.79–46.79 mg GAE/g powder). The present findings allow us to understand the effect of drying methods on the properties of chamomile extract powder and provide a better drying option to dry chamomile extract. Due to higher powder yield with ideal powder properties such as low moisture content and water activity, desirable color, and high total polyphenol content obtained from convection oven-drying, convection oven-drying was a better option than freeze-drying and spray-drying for drying chamomile extract.

## 1 Introduction

Chamomile (*Matricaria chamomilla* L.) is a well-known medicinal plant used by local communities around the world for thousands of years (Kolanos and Stice, 2021). It is used to treat hay fever, inflammation, muscle spasms, menstrual disorders, insomnia ulcers, wounds, gastrointestinal disorders, rheumatic pain, and hemorrhoids (Srivastava et al., 2010). A recent study has shown that presence of chamomile extract in the isotonic seawater spray served as a good alternative treatment option for patients with hay fever (Atar et al., 2022). The main constituents contributing to chamomile’s therapeutic potential are terpenoids (chamazulene and bisabolol) and flavonoids (apigenin, luteolin, and quercetin) (Kolanos and Stice, 2021). Sándor et al. (2018) explained that the smooth muscle relaxant effect of chamomile extract was correlated with the flavonoid content of the extract.

Due to the high initial moisture content of fresh chamomile flowers (83.34 ± 0.7) %, they are considered highly perishable (Rahimi et al., 2014). To increase the shelf life, the fresh chamomile flowers are subjected to drying post-harvest. Dried chamomile flowers are usually sold in whole flowers or ground into tea bags, either in pure or blended with other herbs. Besides that, the chamomile preparations are also marketed in form of infusions, decoctions as well as powder resulting from the drying of the extract. The dried extract in form of powder offers much flexibility for new therapeutic formulations as it could be used as a replacement for liquid extract and serve as a shelf-stable ingredient in new applications. The dried extract also shows greater benefits over its liquid counterpart as it has a higher concentration of bioactive compound and higher storage stability with reduced storage cost required (Goula et al., 2004). Dried extract also allows standardization and quality control of herbal preparation to be done much easier.

Despite several studies on the drying of fresh chamomile flowers and extraction of dried chamomile flowers (Abbas et al., 2021; Abutaleb et al., 2021; Motevali et al., 2016; Molnar et al., 2017; Žlabur et al., 2020), the development of dried chamomile extract for subsequent therapeutic application has not been reported. Drying of liquid extract is a crucial step as it limits enzymatic degradation and microbial growth while preserving the bioactive properties of the extract (Yuan et al., 2015).

Dried chamomile extract can be produced by drying the liquid extract obtained from the dried chamomile flowers. Several drying methods can be used which include convection oven-drying, spray-drying, and freeze-drying. Convection oven-drying is a low-cost drying method. It utilizes hot air as a drying medium by circulating it in the oven and passing it over the moist food to carry moisture away from the food. Freeze-drying involves freezing food followed by subjecting the frozen food under reduced pressure so that ice crystals from food can be sublimed into vapor. This type of drying allows drying to occur under low temperature and oxygen level, which is suitable for heat-labile and oxygen-sensitive food. On the other hand, spray-drying is widely used in commercial for production of powder. During spray-drying, the feed is atomized into fine droplets in stream of hot air. These fine droplets are dried in seconds after contacted with the hot air in the drying chamber and collected in form of powder or granules. Spray-drying is one of the common methods used to microencapsulate polyphenols (Fang and Bhandari, 2012).

Nevertheless, each drying method has its own advantages and limitations. Drying not only reduces the moisture content of extract but also alters other physicochemical properties and bioactivity of extract. This is because drying involves removal of large amount of moisture from extract that could change the final characteristics of dried product. The final characteristics of dried product is extensively affected by the drying method and conditions (Sundaram, 2008). Therefore, the aim of the study is to determine and compare the yield, physicochemical properties, and total polyphenol content of convection oven-dried, spray-dried, and freeze-dried chamomile extract powder. The study also aims to identify the drying method that is most appropriate to produce chamomile extract powder with ideal powder properties at minimal production cost.

## 2 Materials and methods

### 2.1 Materials

Dried whole chamomile flowers (Brand: PWJ Natural Herbs) were purchased from online store (Cheng Woh), Malaysia. Food grade modified corn starch (Brand: CLEARAM® CH 20) was purchased from online store (Online Mart Penang), Malaysia. Gallic acid (≥98.0% purity), Folin reagent (Analytical reagent grade), sodium carbonate (Analytical reagent grade), and methanol (Analytical reagent grade) were purchased from Scienfield Expertise PLT, Selangor, Malaysia.

### 2.2 Methods

#### 2.2.1 Preparation of chamomile extract

Pure chamomile extract was prepared by steeping 10 g of dried chamomile flowers in 200 ml distilled water at 80°C for 15 min, adapted from (Osman et al., 2016). The extract was then filtered using a Buschner filter to remove the solid particulates. The total solid content of the extract was determined using the method as shown in Section 2.2.3.1. Extract was then added with 6 g of modified corn starch as the carrier. The proportion of carrier to extract was based on the total solid content of feed that allowed successful powder formation obtained from spray-drying. The mixture was described as liquid feed and dried using different drying methods as shown in Section 2.2.2.

#### 2.2.2 Drying methods

##### 2.2.2.1 Convection oven-drying

Liquid feed (200 ml) was poured into steel tray and dried in a convection oven (Memmert UF110, Germany) at 45°C until a constant dry mass was obtained (Shuen et al., 2021). The dried sample was scraped into flakes and crushed into powder using a food processor (Panasonic Smart Food Processor MK-F800SSL, Japan).

##### 2.2.2.2 Spray-drying

Liquid feed (200 ml) was spray-dried using spray dryer (Buchi Mini Spray Dryer B-290, Germany) at an inlet temperature of 140°C at two different levels of feed flow rate (10.5 ml/min and 12 ml/min). The temperature and feed flow rates were selected according to the minimum process conditions tested using the same model of spray dryer in a study conducted by Santiago-Adame et al. (2015). The spray-dried powder was collected at collection vessel and weighed.

##### 2.2.2.3 Freeze-drying

Liquid feed (200 ml) was frozen overnight at −80°C in a freezer. The frozen sample was then placed in a freeze-dryer (Labconco FreeZone freeze dryer, United States) to be dried for 72 h at −50°C at 0.500 mbar, adapted from (Phing et al., 2022). The dried sample was weighed, scraped into flakes, and subsequently ground into powder using a food processor (Panasonic Smart Food Processor MK-F800SSL, Japan).

All chamomile extract powder samples obtained from Section 2.2.2.1, Section 2.2.2.2, and Section 2.2.2.3 were packed into aluminium foil bag and kept in room temperature for further analysis.

#### 2.2.3 Analysis

##### 2.2.3.1 Total solid content

Total solid content of chamomile extract was measured by heating the extract in a convection oven (Memmert UNB500, Germany) at 105 °C until a constant dry mass was obtained. The mass of the extract before and after drying were weighed and recorded.

Total solid content of chamomile extract was calculated using Eq. 1:

$$Total\;solid\;content=\frac{m_{2}}{m_{1}}\;x\;100\%$$ (1)

Where m_2_ is the mass of the dried chamomile extract (g) and m_1_ is the mass of chamomile extract before drying (g).

##### 2.2.3.2 Powder yield (%)

Powder yield (%) of each drying method was calculated using Eq. 2:

$$Powder\;yield\;\left(\%\right)=\frac{Mass\;of\;powder\;obtained\;\left(g\right)\;}{Mass\;ofdried\;chamomile\;extract+modified\;corn\;starch\;\left(g\right)}x\;100\%$$ (2)

##### 2.2.3.3 Physicochemical properties

###### 2.2.3.3.1 Moisture content (%)

Chamomile extract powder samples obtained from different drying methods were dried overnight in convection oven (Memmert UNB500, Germany) at 105°C until the powder samples were dried to constant mass. Mass of powder before and after drying were measured to calculate moisture content using Eq. 3:

$$Moisture\;content\left(\%\right)=\frac{Mass\;of\;powder\;before\;drying\;\left(g\right)\;–\;Mass\;of\;powder\;after\;drying\left(g\right)}{Mass\;of\;powder\;before\;drying\;\left(g\right)}\;x\;100\%$$ (3)

###### 2.2.3.3.2 Color

Colour of chamomile extract powder samples obtained from different drying methods were measured using a colorimeter (HunterLab, Colorflex EZ Colorimeter, United States) and expressed as L*(lightness), a* (redness), and b*(yellowness) values.

###### 2.2.3.3.3 Water activity

Water activity of chamomile extract powder samples obtained from different drying methods were measured using a benchtop water activity meter (METER Aqualab 4 TE, United States). Powder samples were placed in sample cups and placed in the sample port for reading.

##### 2.2.3.4 Total polyphenol content

Total polyphenol content of chamomile extract powder samples obtained from different drying methods were measured using Folin-Ciocalteu method as described by Ismail et al. (2004). Gallic acid was used as standard. 0.5 mg/ml gallic acid stock standard solution was prepared by dissolving 250 mg of gallic acid in 1 ml methanol, and diluted to 500 ml of distilled water. Working standards of between 0.01 and 0.05 mg/ml were prepared by diluting the gallic acid stock solution with distilled water. The extract was prepared at concentration of 1 mg/ml by dissolving 1 mg extract powder in 1 ml distilled water. 100 µL of extract/standard was transferred into a test tube and 0.75 ml of Folin-Ciocalteu reagent (previously diluted 10-fold with distilled water) was added and mixed. The mixture was allowed to stand at room temperature for 5 min. Then, 0.75 ml of 6% (w/v) sodium carbonate was added to the mixture and mixed. The mixture was allowed to stand for another 90 min under dark condition at room temperature. Sample and standard absorbance were then read at 725 nm using a PRIM light spectrophotometer (Aqualabo, France). The standard calibration curve of gallic acid (0.01–0.05 mg/ml) was plotted and used to determine total polyphenol content of extract powder. The total polyphenol content of extract powder was expressed as gallic acid equivalent (GAE) in mg per g powder.

##### 2.2.3.5 Statistical analysis

All analysis was conducted in duplicates. Results were presented as mean ± SD. Data were analysed using IBM SPSS Statistics (RRID:SCR_016479). A one-way analysis of variance (ANOVA) and Tukey’s test were used to establish the significance of difference (*p* < 0.05) between different drying methods.

## 3 Results

### 3.1 Visual examination of chamomile extract powder

**FIGURE 1:**
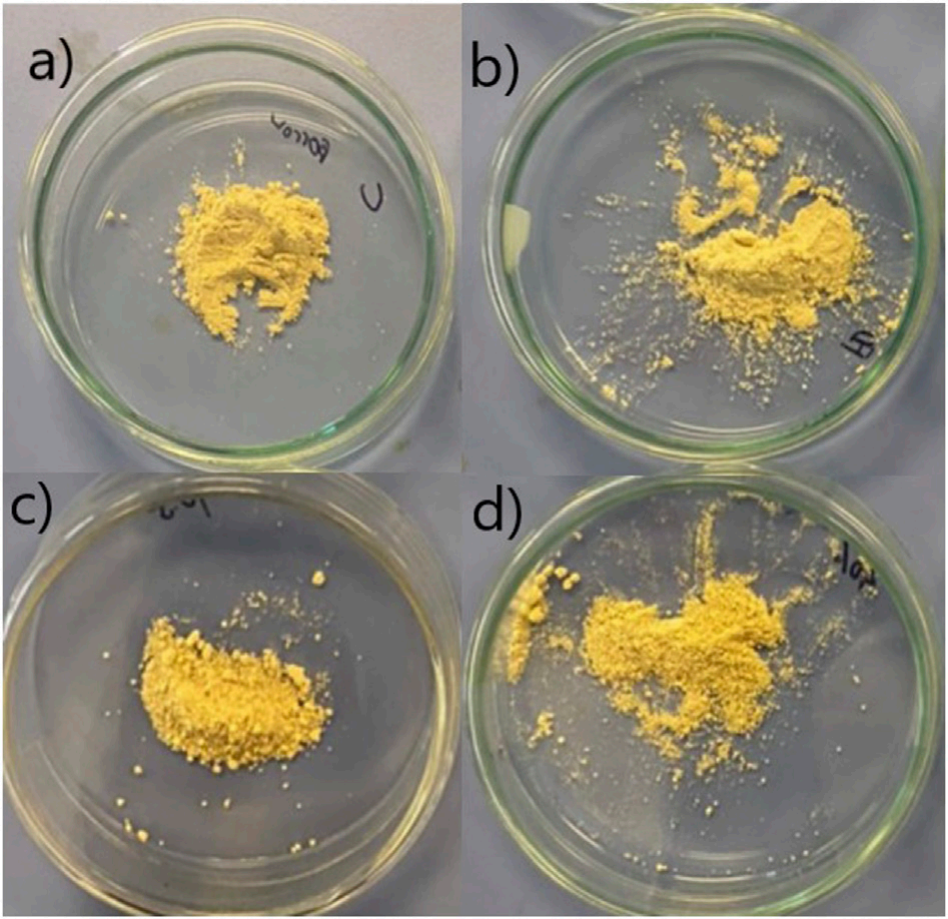
Chamomile extract powder obtained from convection oven-drying a), freeze-drying b), and spray-drying at 140°C, 10.5 ml/min c), and spray-drying at 140°C, 12 ml/min d).

The convection oven-dried, freeze-dried, and spray-dried chamomile extract powder obtained from our study were yellowish as shown in Figure 1.

Based on Figure 1, it could be seen that convection oven-dried and freeze-dried chamomile extract powder were lighter in color and finer in size compared to those spray-dried. It was also observed that some particles of the spray-dried chamomile extract powder were partially agglomerated.

### 3.2 Powder yield

Table 1 shows the chamomile extract powder yield obtained from various drying methods. Based on Table 1, it could be seen that chamomile extract powder yield obtained from convection oven-drying was the highest (90.17%), followed by freeze-drying (83.24%), spray-drying at 140°C, 10.5 ml/min (26.99%), and spray-drying at 140°C, 12 ml/min (16.67%).

**TABLE 1:** Powder yield of convection oven-drying, freeze-drying and spray-drying.

Drying methods	Powder yield (%)
Convection oven-drying	90.17 ± 5.56^a^
Freeze-drying	83.24 ± 0.16^a^
Spray-drying at 140°C, 10.5 ml/min	26.99 ± 3.35^b^
Spray-drying at 140°C, 12 ml/min	16.67 ± 0.00^b^

Notes: Each value in the table represents the mean ± standard deviation (SD) with *n* = 2. Mean ± SD, followed by different superscript letters within the same column are significantly different at *p* ≤ 0.05.

### 3.3 Physicochemical properties

The chamomile extract powder obtained from different drying methods were characterized by the physicochemical properties such as moisture content, color attribute, and water activity. Table 2 shows the effect of drying methods on physicochemical properties of chamomile extract powder.

**TABLE 2:** Physicochemical properties of chamomile extract powder obtained from different drying methods.

Drying methods	Moisture content (%)	Colour			Water activity
		L*	a*	b*	
Convection oven-drying	8.50 ± 0.70^ab^	80.76 ± 0.08^ab^	2.46 ± 0.17^a^	25.25 ± 0.81^a^	0.52 ± 0.02^a^
Freeze-drying	7.50 ± 0.70^a^	82.72 ± 0.61^a^	1.35 ± 0.01^a^	20.17 ± 0.18^b^	0.63 ± 0.00^b^
Spray-drying at 140°C, 10.5 ml/min	11.00 ± 1.40^b^	74.42 ± 3.55^b^	4.21 ± 0.67^b^	29.70 ± 0.98^c^	0.57 ± 0.04^c^
Spray-drying at 140°C, 12 ml/min	12.00 ± 0.00^b^	77.92 ± 0.02^ab^	3.86 ± 0.06^b^	30.51 ± 0.13^c^	0.58 ± 0.00^c^

Notes: Each value in the table represents the mean ± standard deviation (SD) with *n* = 2. Mean ± SD, followed by different superscript letters within the same column are significantly different at *p* ≤ 0.05.

Based on Table 2, it could be observed that the moisture content of chamomile extract powder obtained from different methods was ranged from 7.50% to 12.00%. In terms of water activity, freeze-dried chamomile extract powder was found to have the highest water activity (0.63) compared to those convection oven-dried (0.52), spray-dried at 140°C, 10.5 ml/min (0.57), and spray-dried at 140°C, 12 ml/min (0.58). Color of powder is an important attribute as it reflects its sensory attractiveness. In this study, the chamomile extract powder obtained from different drying methods were all yellowish in color by visual perception as shown in Figure 1. Both convection oven-dried and freeze-dried chamomile extract powder were found to have light yellowish color, which is in accordance with the L* values determined for the powder samples. L* represents the lightness and darkness of color where 0 indicates black and 100 indicates white. The higher the L* value, the lighter the color of the sample. The positive a* value indicates the degree of redness (0–60) while the negative a* value indicates the degree of greenness (0 to −60) of powder. As shown in Table 2, both spray-dried chamomile extract powder samples were found to have higher a* values (3.86–4.21). The positive b* value indicates the degree of yellowness (0–60) while the negative b* value indicates the degree of blueness (0 to −60). The b* values of both spray-dried chamomile extract powder samples were also higher (29.70–30.51).

### 3.4 Total polyphenol content

Since chamomile is a source of polyphenols with primarily flavonoids, the total polyphenol content of chamomile extract powder was also determined in our study. Table 3 shows the total polyphenol content of all chamomile extract powder obtained from various drying methods.

**TABLE 3:** Total polyphenol content of chamomile extract powder obtained from different drying methods.

Drying methods	Total polyphenol content (mg GAE/g powder)
Convection oven-drying	56.94 ± 5.98^a^
Freeze-drying	55.98 ± 7.33^a^
Spray-drying at 140°C, 10.5 ml/min	42.79 ± 3.98^a^
Spray-drying at 140°C, 12 ml/min	46.79 ± 14.87^a^

Notes: Each value in the table represents the mean ± standard deviation (SD) with *n* = 2. Mean ± SD, followed by different superscript letters within the same column are significantly different at *p* ≤ 0.05.

Among the different powder samples, both spray-dried chamomile extract powder samples were found to have the lowest total polyphenol content (42.79–46.79 mg GAE/g powder).

## 4 Discussion

### 4.1 Visual examination of chamomile extract powder

The agglomeration of spray-dried chamomile extract powder may be due to its higher residual moisture as evidenced by determination of its moisture content (11.00%–12.00%). Compared to the brownish commercial dried chamomile flower samples shown in a study conducted by Catani et al. (2021), our chamomile extract powder obtained from different drying methods achieved vivid yellowish color, as all the dust and impurities were filtered from the extract before proceed to the drying process.

### 4.2 Powder yield

Powder yield obtained from convection oven-drying was the highest as the drying process itself did not involve many processing steps that could lead to loss of yield as compared to freeze-drying and spray-drying. Due to low density and high porosity of freeze-dried chamomile extract powder, it affected the powder flowability and some of the powder was lost during manual transfer. The lowest powder yield obtained from spray-drying was due to significant amount of fine powder being deposited on the drying chamber wall during the spray-drying process. By sticking to the chamber wall, less powder was collected from the separating cyclone and hence the powder yield decreased. Higher feed flow rate in spray-drying could also lower temperature within spray-dryer, resulting in insufficient drying of droplets and visible depositions of droplets/particles on the chamber wall. This explains the decrement of powder yield from 26.99% to 16.67% when the spraying feed flow rate increased from 10.5 ml/min to 12 ml/min. Decrease in feed flow rate from 12 ml/min to 10.5 ml/min resulted in increase of powder yield to 26.99% as it implies a longer contact time between feed and hot drying air which allows hear transfer and water evaporation to occur more efficiently (Phisut, 2012). Our finding agree with study conducted by Shuen et al. (2021) on drying of *kuini* extract and study conducted by Gopinathan et al. (2020) on drying of *cempedak*.

### 4.3 Physicochemical properties

The range of moisture content of the chamomile extract powder (7.50%–12.00%) obtained was quite similar to the *cempedak* and oyster mushroom powder from a study conducted by Rohmah et al. (2022). Among the chamomile extract powder samples, moisture content of spray-dried chamomile extract powder (11%–12%) was higher compared to those convection oven-dried (8.50%) and freeze-dried (7.50%). This is in contradiction to finding obtained by Shuen et al. (2021) where they reported the lowest moisture content of spray-dried *kuini* powder compared to those freeze-dried and convection oven-dried. The lower moisture content reported for the spray-dried powder could be due to lower feed flow rate (10 ml/min) and higher spray-drying temperature applied (170°C) in their study. Besides, the high moisture content of spray-dried chamomile extract powder could be explained by amorphous nature of modified corn starch and rapid spray-drying process that caused the dried powder to develop into complete amorphous state that tends to absorb high amount of moisture during powder handling process after spray-drying (Azhar, 2021). The higher feed flow rate in spray-drying also led to an increase in moisture content of chamomile extract powder as could be seen in Table 2. This can be attributed to shorter contact time between extract and drying air, making the heat transfer less efficient and resulting in lower water evaporation (Carmona et al., 2018). Both convection oven-dried and freeze-dried chamomile extract powder displayed no significant difference in moisture content, in accordance to finding obtained by Phing et al. (2022).

It has been reported that powder moisture content below 10% is considered microbiologically safe (Tze et al., 2012). Since the spray-dried chamomile extract powder obtained from two different spraying conditions were having moisture content higher than 10%, these powder samples may not achieve microbial safety. The higher residual moisture found in these two samples could have resulted in particle agglomeration as observed in our Figure 1.

Freeze-dried chamomile extract powder was found to have the highest water activity (0.63) compared to those convection oven-dried (0.52), spray-dried at 140°C, 10.5 ml/min (0.57), and spray-dried at 140°C, 12 ml/min (0.58). Water activity of powder is significantly affected by drying method which in a study conducted by Caliskan and Dirim (2016) has shown that freeze-dried sumac extract powder had a higher water activity than spray-dried sumac extract powder. Higher water activity means there would be higher amount of free water that is available in powder for microbial growth. Since bacteria, yeast, and mold growth are inhibited below water activity of 0.6 (Gobbetti et al., 2010), freeze-dried chamomile extract powder with water activity higher than 0.6 could be highly susceptible to microbial growth. All chamomile extract powder samples obtained in our study with water activity above 0.40 were considered not stable for chemical and enzymatic reactions (Marques et al., 2007; Quek et al., 2007).

The lighter color of convection oven-dried (80.76) and freeze-dried chamomile extract powder (82.72) could be due to limited color degradation under low drying temperature. The lower L* values of both spray-dried chamomile extract powder samples (74.42–77.92) indicate that they were darker in color compared to the other powder samples. The darker color observed in spray-dried powder could be due to exposure of high temperature during spraying process that leads to Maillard browning. The temperature used for spray-drying was 140°C while temperature used for convection oven-drying and freeze-drying were only 45°C and −50°C, respectively. Moreover, caramelization could also occur due to high temperature during spray-drying which contributes to darkening after drying (Caparino et al., 2012). As shown in Table 2, both spray-dried chamomile extract powder samples were found to have higher a* values (3.86–4.21) than those convection oven-dried and freeze-dried (1.35–2.46). This indicates that both spray-dried chamomile extract powder samples were slightly redder in color compared to those convection oven-dried and freeze-dried. The higher b* values (29.70–30.51) of both spray-dried chamomile extract powder samples than those convection oven-dried and freeze-dried (20.17–25.25) means that they were more yellowish in color. These results revealed that higher drying temperature in spray-drying (140 °C) could lead to darker, redder, and more yellowish powder that could be caused by heat-induced Maillard reaction and caramelization (Koca et al., 2015).

### 4.4 Total polyphenol content

The lower amount of total polyphenol content found in spray-dried chamomile extract powder could be attributed to high temperature in spray-drying that led to higher degree of polyphenol degradation (Shuen et al., 2021). The phenolic compounds have been reported to be highly sensible to temperature, therefore they are easily decomposed when exposed to severe heat treatments (Poonam, 2014). Since lower drying temperature were used in both convection oven-drying and freeze-drying, there were higher retention of total polyphenol content in powder. High residual moisture found in spray-dried chamomile extract powder could also lead to further degradation of phenolic compounds (Alean et al., 2016). Based on a recent study conducted by Catani et al. (2021), the total polyphenol content for dried chamomile extract ranged from 41.1 to 100.5 mg GAE/g dried extract. Direct comparison in terms of total polyphenol content could not be made within this study as the measurement of total polyphenol content was based on total mass of powder which included both extract and drying aid.

The findings of this study have to be seen in light of some limitations. Although the total polyphenol content of chamomile extract powder obtained from different drying methods were determined, further research is needed to confirm the identity and quantity of phenolic compounds found in the chamomile extract powder. Individual phenolic compounds may have different stability towards different storage conditions of different temperature and water activity. For similar considerations, the storage stability of polyphenol compound in the chamomile extract powder has to be conducted. Nevertheless, it cannot be ruled out that drying methods did affect the powder yield, physicochemical properties (moisture content, water activity, and color attributes), and total polyphenol content of chamomile extract powder, as shown in this study.

## 5 Conclusion

Spray-drying conducted at 140°C, 10.5 ml/min, and 140°C, 12 ml/min had resulted in lower powder yield, higher moisture content and water activity of powder. Due to higher moisture content, partial agglomeration was also observed in spray-dried chamomile extract powder. High temperature during spray-drying also led to undesirable color and higher degradation of extract polyphenol. Freeze-drying resulted in production of chamomile extract powder with higher water activity that could be susceptible to microbial growth. Our study showed that the convection oven-drying was the most appropriate drying method for drying of heat-sensitive chamomile extract. Convection oven-drying allowed higher powder yield, lower moisture content and water activity, desirable color as well as higher total polyphenol content of chamomile extract powder.

## Data Availability

The raw data supporting the conclusion of this article will be made available by the authors, without undue reservation.
